# Tactile stimulation of young WAG/Rij rats prevents development of depression but not absence epilepsy

**DOI:** 10.3389/fnbeh.2024.1433431

**Published:** 2024-06-27

**Authors:** Aymen Balikci, Ugur Eryilmaz, Vildan Keles Guler, Gul Ilbay

**Affiliations:** Department of Physiology, Faculty of Medicine, Kocaeli University, İzmit, Türkiye

**Keywords:** absence epilepsy, tactile stimulation, WAG/Rij rats, depression, adolescence

## Abstract

Investigations in Wistar Albino Glaxo from Rijswijk (WAG/Rij) rats that are susceptible to genetic absence epilepsy have demonstrated that environmental modifications affect absence seizures. Previously, we showed that neonatal tactile stimulations produce disease-modifying effect on genetically determined absence epilepsy and associated depression in Wag/Rij rats. The study presented here examined the effect of TS during late ontogenesis (adolescence and young adulthood) on epilepsy and depression outcomes in this genetically epileptic rat strain. On postnatal day (PND) 38, male WAG/Rij rats randomly were assigned to either the tactile stimulation (TS), handled or control group (unhandled) with 8 animals in each group. Following a 7-day adaptation period to their new surroundings, the animals were submitted to tactile stimulation from PND 45 to PND 90, five days per week, for 5 min daily. The tactile-stimulated rat was removed from its cage, placed on the experimenter’s lap, and had its neck and back gently stroked by the researcher. The handled rats were taken to another cage and left alone for 5 min daily from PND 45 to PND 90. The control rats were left undisturbed in their home cage, except for regular cage cleaning. After PND 90, all rats were left undisturbed until behavioral testing and EEG recording. When the animals were 7 months old, they were subjected to the sucrose consumption test (SCT) and the forced swimming test (FST). Electroencephalogram (EEG) recordings were made at 8 months of age in order to measure electroencephalographic seizure activity, thus, the spike–wave discharges (SWDs). Tactile-stimulated rats showed increased sucrose consumption and number of approaches to the sucrose solution in the SCT when compared with the handled and control rats. In the FST, rats in TS group showed lower immobility time and greater immobility latency, active swimming time and diving frequency than the handled and control rats. The duration and the number of seizures were not different amongst the groups. The data obtained suggest that TS in young rats is able to prevent depression in WAG/Rij rats.

## Introduction

Epilepsy is a group of progressive neurologic conditions that are characterized by spontaneous recurrent seizures and affects about 1% of the population. In patients with epilepsy, seizure activity is suppressed symptomatically with medical treatment, but they are not disease-modifying as they have no effects on the mechanisms of the brain to generate seizures (Dezsi et al., 2013).

Treatments are also not effective for the psychiatric comorbidities observed in individuals with epilepsy, including anxiety, depression, and psychosis, which add to the disability burden (Vega et al., 2011; Dezsi et al., 2013; Dagar and Falcone, 2020; Holmes, 2021).

Translational epilepsy research not only aims to identify symptomatic treatments; but also, to modify the disease (Galanopoulou et al., 2012; Brock et al., 2021).

Recent experimental reports have demonstrated that there are successful therapies that have interfered with epileptogenesis (Blumenfeld et al., 2008; Russo et al., 2010; Wong, 2010; McClelland et al., 2011; Balikci et al., 2020; Ilbay et al., 2022; Sarkisova and van Luijtelaar, 2022). Moreover, recent evidence for the effects of early life manipulations on the epileptic and behavioral phenotype of WAG/Rij rats supports the disease-modifying treatment notion (Sarkisova and Gabova, 2018; Balikci et al., 2020; Ilbay et al., 2022; Sarkisova and van Luijtelaar, 2022).

The WAG/Rij rat strain is a genetic model of absence epilepsy with depression-like comorbidity (Coenen and Van Luijtelaar, 2003; Sarkisova and van Luijtelaar, 2011; Sarkisova and van Luijtelaar, 2022). The animals of this strain are born without seizures. Seizures and their EEG hallmarks, SWDs, appear at the age of 2 months and reach a maximum at the age of 7–9 months, when the number of daily SWDs is approximately 16 to 18 per hour, the frequency is about 7–11 Hz, and the mean duration is about 5 s (Coenen and Van Luijtelaar, 1987; Sarkisova et al., 2010; Sarkisova and van Luijtelaar, 2022; Zhuravlev et al., 2022). Recent experimental research indicates that improvements of maternal care in the early stages of development can impact the progression of genetically determined absence epilepsy with depression (Sarkisova and Gabova, 2018; Sarkisova and van Luijtelaar, 2022).

Depressive WAG/Rij rat mothers show reduced maternal grooming and licking behavior toward their own pups as compared to normal Wistar rat mothers (Dobryakova et al., 2008; Sarkisova and Gabova, 2018). If WAG/Rij rats were raised by foster Wistar mothers who provided them with good maternal care, they had fewer SWDs and shorter durations of comorbid depression (Sarkisova and Gabova, 2018).

Maternal care during early life provides a high level of tactile stimulation and reduces the manifestation of adult pathologic phenotype in WAG/Rij rats. This is achieved through alterations in the activity of DNA methyltransferases, which are enzymes responsible for catalyzing DNA methylation and generating epigenetic modifications linked to gene transcriptional repression. Changes in DNA methylation status caused by maternal care may hinder the decrease in hyperpolarization-activated cation current and the expression of HCN1 ion channel in the somatosensory cortex, which is responsible for the occurrence of SWDs. Additionally, it may lead to a decrease in the functioning of the mesolimbic dopaminergic brain system, which is responsible for the behavioral symptoms of depression (Sarkisova and van Luijtelaar, 2022).

In a study done by our group we found that neonatal tactile stimulation slows down the development of absence epilepsy and comorbid depression-like symptoms in adult WAG/Rij rats (Balikci et al., 2020). In our next study, we have shown that tactile stimulations during the early postnatal period have a long-term impact on dendrite structure in WAG/Rij rat’s brain, and neonatal tactile stimulation can regulate dendritic spines in layer V in pyramidal neurons of SoCx which previously defined as the focus of epilepsy (Ilbay et al., 2022). Although there are exciting results showing the positive effects of enriching tactile experiences in early life on absence epilepsy and accompanying depression-like behaviors in WAG/Rij rats, research on the effects of tactile experiences later in life does not exist.

Throughout their lifespan, rats exhibit affiliative activities that provide them with rich tactile stimulation.

Allogrooming is a common social behavior observed in wild rats when one rat licks or nibbles the fur of another rat of the same species (Schweinfurth, 2020). In wild rats, the act of allogrooming is typically focused on the areas of the body that are difficult to access, such as the face or the neck (Schweinfurth et al., 2017; Schweinfurth, 2020). Rats experience grooming from their mothers throughout infancy. Subsequently, infants extend this behavior to other members of the colony (Sachs,1988; Schweinfurth et al., 2017; Schweinfurth, 2020).

It is not known how much time WAG/Rij rats spend for allogrooming. It can be expected that the poor maternal care may have an impact on the affiliative behaviors of WAG/Rij rats.

As a result of the poor maternal care, a decrease in affiliative behaviors and tactile stimulations can occur in WAG/Rij rats later in life (Dobryakova et al., 2008, 2011).

Adolescence and young adulthood in humans are two periods where interactions with peers become especially important. These relationships provide valuable insights into behavioral patterns, attitudes, and beliefs (Paus, 2013; Lam et al., 2014; Clark et al., 2023). Adolescence is characterized by increased peer interactions compared to other stages of development. These interactions are an important source of tactile stimulation (Field, 2014; Lam et al., 2014; Della Longa et al., 2022).

Similarly, adolescent rats are engaged in more social interactions compared to both younger and older animals (Spear and Brake, 1983; Trezza et al., 2011). The social activity in adolescent and young rodents is crucial to express and understand communication signals (Vanderschuren et al., 1997; Trezza et al., 2011). Furthermore, during adolescence and young adulthood, rich social interaction serves as the main source for tactile experiences based on social contact (Field, 2014).

Tactile stimulation is a form of social contact enrichment that improves sensory stimulation and results in neurobiological alterations that decrease anxiety and improve cognition and memory in rats (Richards et al., 2012; Antoniazzi et al., 2014; Balikci et al., 2020; Ilbay et al., 2022). Studies have shown that tactile stimulation promotes changes in the brain’s structure and function, which helps to alleviate the negative effects of pathological processes (Gibb et al., 2010; Richards et al., 2012; Costa et al., 2020; Ilbay et al., 2022).

Multiple neurobehavioral, morphological, neurochemical, and pharmacological findings indicate that the brain continues to develop during adolescence and young adulthood (Cunningham et al., 2002; Steinberg, 2005; Tamnes et al., 2010; Wahlstrom et al., 2010; Lebel and Beaulieu, 2011; Arain et al., 2013; Schneider, 2013; Larsen and Luna, 2018). Tubulinogenesis, axonogenesis, and synaptogenesis take place during the time before birth and shortly after birth, while myelinogenesis continues into adolescence and early adulthood.

Likewise, glutamatergic neurotransmission is completed during the period before and immediately after birth, whereas the construction of GABAergic neurotransmission in the prefrontal cortex continues (Li and Xu, 2008; Arain et al., 2013). Brain development continues into the early adulthood (Yakovlev and Lecours, 1967; Benes, 1989). Studies have indicated that the progressive myelination, synaptic pruning, and sprouting of mesocortical dopamine fibers play a crucial role in the development of cognitive abilities and emotional regulation during the late adolescence and early adulthood (Cunningham et al., 2002; Arain et al., 2013; Larsen and Luna, 2018).

In this light, with this study, we aimed to determine the effect of a supportive environment, that is TS, during the late adolescence and early adult period on absence epilepsy and comorbid depression-like behaviors of WAG/Rij adult rats.

## Materials and methods

### Animals

The experimental procedures were approved by the University of Kocaeli Animal Ethics Committee in agreement with the guidelines for the Care and Use of Animals for Scientific Purposes (KOU HADYEK 7/3–2021).

The WAG/Rij rats were maintained in a 12-h light/dark cycle (lights on at 7:00 a.m.) and had unrestricted access to food and water. Male WAG/Rij rats were selected in order to eliminate the influence of hormonal cycles in females as a variable. On the 38th day after birth, male WAG/Rij rats were randomly divided into three groups: the tactile stimulation (TS) group, the handled group, and the control group (unhandled). Each group consisted of 8 animals. The rats were placed in cages with four rats per cage and allowed to adapt to their environment for a period of 7 days prior to the initiation of the TS intervention.

**Figure 1:**
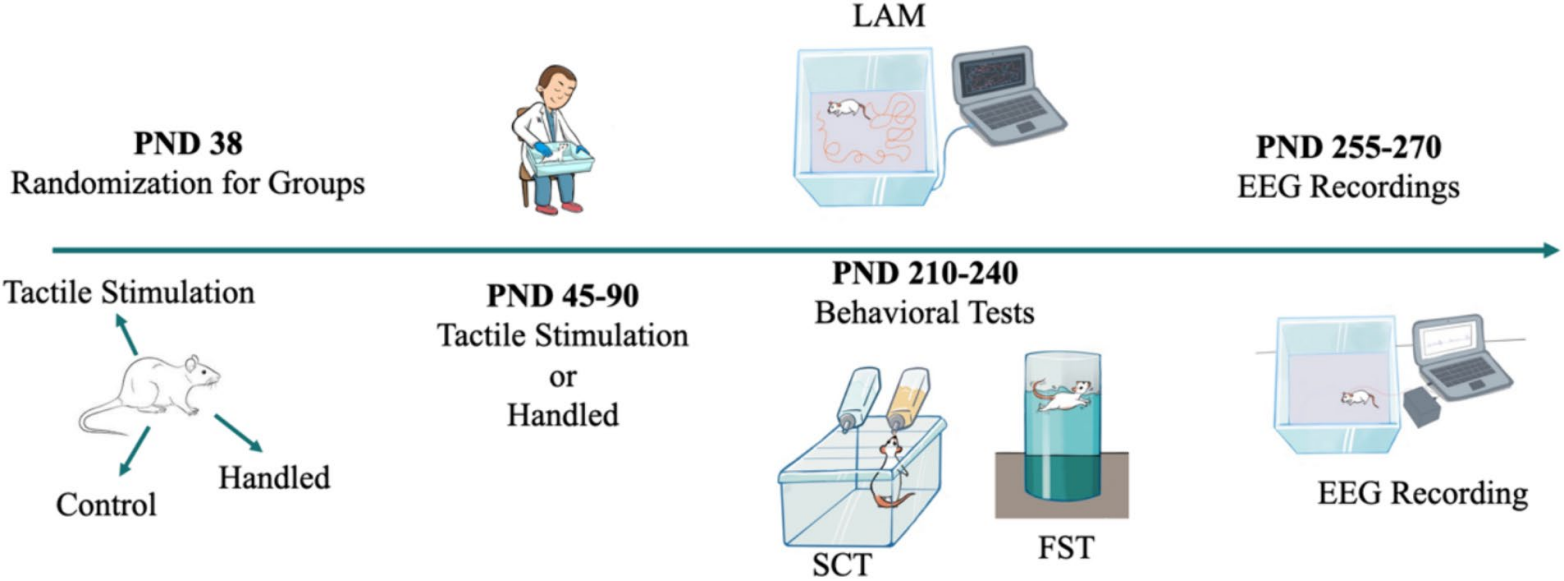
Experimental design. WAG/Rij rats were randomly divided into the tactile stimulation group, the handled group, and the control group (PND 38). Tactile stimulation and handling (removing the animals from their original cages and placing them in a separate cage for a duration of 5 min) were submitted (PND 40–90). Behavioral testing (LAM: Locomotor Activity Monitoring; SCT: Sucrose Consumption Test; FST: Forced Swimming Test) was applied (PND 210–240), and subsequently, EEG recording was conducted (PND 255–270).

The animals had tactile stimulation from adolescence (postnatal day [PND 45]) to adulthood (PND 90). The technique was consistently implemented by the same researcher, daily from Monday to Friday, between the hours of 12 p.m. and 4 p.m., for a duration of 7 weeks. The tactile stimulation involved daily sessions of 5 min, during which the animal was taken out of its cage and placed on the researcher’s lap. The researcher then gently caressed the animal’s neck and back (Costa et al., 2020). In contrast to the TS method, the handling technique entails removing the animals from their original cages and placing them in a separate cage for a duration of 5 min (Costa et al., 2020). The Control WAG/Rij rats were confined to their home cage and were only interacted with during the routine cleaning of the cage. After PND 90, all rats were maintained under normal conditions until the time of behavioral testing and EEG recordings. Behavioral testing and EEG recordings were performed in adulthood (7–8 months) (Figure 1).

### Behavioral tests

All WAG/Rij rats (*n* = 8 for control, *n* = 8 for handled, *n* = 8 for TS intervention) were individually exposed to a forced swimming test (FST), sucrose consumption test (SCT) and locomotor activity test at the age of 7 months between 10:00 am and 4:00 pm each day of the tests.

### Forced swimming test

The depressive-like behaviors are assessed using the Forced Swimming Test (FST), which has been utilized in numerous researchers with slight variations. The experiment took place in a clear cylinder with dimensions of 47 × 38 cm, which was filled with water at a temperature of 22 ± 1°C. At the beginning, rats were made to swim for 15 min during the pretest session and were subsequently dried before being returned to their cages. Following a 24-h interval from the pretest session, rats were subjected to the forced swimming test once more, this time for a duration of 5 min. Their swimming behavior was recorded using video cameras. Then, an impartial evaluator assessed the duration of inactive swimming (immobility), the time it took for immobility to occur (immobility latency), and the overall amount of active swimming (swimming time) for each rat. Immobility is the state of being completely still, with no other movement save for the necessary action of keeping the head above water (Balikci et al., 2020; Ilbay et al., 2022).

### Sucrose consumption test

The sucrose consumption test (SCT) was used to evaluate anhedonia and motivation. SCT was carried out in a cage that resembled the rats’ home cage. Rats were given access to two bottles, with one containing sucrose solution (20%) and the other containing tap water. The consumption of a sucrose solution and the count of interactions with the bottle were measured for each rat over a period of 15 min. The bottles were weighed prior to and after the test to quantify the sugar consumption. The spilled amount was determined by placing the bottles in an empty cage and then subtracted from the total difference calculated for each rat.

No animals were subjected to any form of food or water deprivation. The values of sucrose consumption on the 4th day were utilized to assess the disparities across rat groups following a 3-day adaption technique, as outlined in prior research. The animals’ hedonic-like states were measured by sucrose consumption, while the number of approaches served as an indirect sign of exploratory activity during the test (Balikci et al., 2020; Ilbay et al., 2022).

### Locomotor activity test

**Figure 2:**
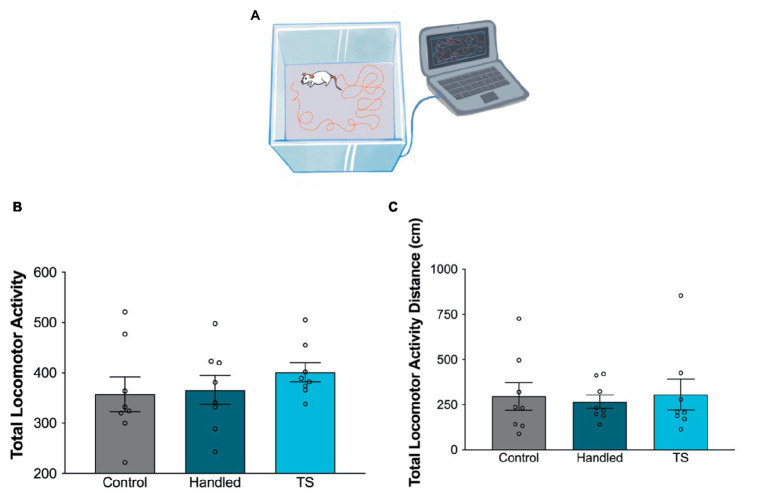
Behavioral evaluations of locomotor activity in the control group, handled and TS given WAG/Rij rats. **(A)** Simplified illustration of rat locomotor activity monitoring. **(B)** Total locomotor activity; **(C)** Locomotor activity-distance. There was no statistical difference between groups (*p* > 0.05).

Locomotor activity was measured using the rat activity monitoring system (Commat Ltd., Ankara, Turkey), which included a Plexiglas test chamber, computer, and activity software (Figure 2A). The rats were transported to the testing room, were acclimatized for a duration of 1 h and were subsequently placed within a plexiglass chamber measuring 42 × 42 × 30 cm. The chamber had infrared photocells consisting of pairs of 15 infrared photobeams and detectors. These were positioned at intervals of 2.5 cm in the horizontal plane (bottom) and 4.5 cm in the vertical plane (upper). Locomotor activity was recorded and analyzed for a duration of 10 min (Balikci et al., 2020; Ilbay et al., 2022). Locomotion was determined by measuring the total distance traveled, and anxiety-related behaviors were evaluated using total locomotor activity (Karson et al., 2012; Miller et al., 2021).

### Surgery, EEG recordings and assessment of absence seizures

At the age of 8 months, 7 animals in each group were equipped with electrodes.

During the stereotaxic surgery, tripolar electrodes (MS3333/2A; Plastic One, the United States) were placed on certain cortical areas to record maximal SWD activity. The frontal electrode was positioned at AP 2.0 mm and L 3.5 mm, the parietal electrode at AP −6.0 mm and L 4.0 mm, and the cerebellar cortex served as the reference electrode. Epidural electrodes were implanted in a small separate circular opening in the skull (diameter = 1–2 mm). Electrode sets were mounted on the skull with a crown of dental cement and fixed with embedded mounting screws (Sitnikova and Van Luijtelaar, 2007). The surgery was performed under Xylazin (5 mg/kg ip) and Ketamine (60 mg/kg ip) anesthesia. Following a two-week period of healing, the animals were connected to the MP150 EEG recording system. After they were habituated to the conditions for 1 h, the EEG recording was conducted for 4 h (between 10:00 am and 2:00 pm). Spike wave discharges (SWD) were examined, which met particular criteria: (Van Luijtelaar and Coenen, 1986; Sitnikova and Van Luijtelaar, 2007) a length of 1–10 s, spike and wave frequencies of 7–10 Hz, and doubled amplitude to background activity.

### Statistical analyses

All statistical analyses were performed in GraphPad Prism 10.2.3 version (United States) statistics program. Data are shown as mean ± SEM. Kolmogorov–Smirnov test was used to assess the assumption of normality. One-Way ANOAVA followed by *post hoc* Tukey’s test was used to compare groups. When the normality assumption did not hold, Kruskal-Wallis test was used to compare the groups. Dunn’s test was used for the multiple comparisons. A *p*-value less than 0.05 was considered to be statistically significant.

## Results

### Behavioral tests

There was no statistically significant difference amongst TS-treated, handled and control WAG/Rij rats in total locomotor activity and distances traveled (*p* > 0.05) (Figures 2B,C). TS or handled WAG/Rij rats did not display different levels of anxiety-like behavior compared to the control group, as assessed by the locomotor activity test.

**Figure 3:**
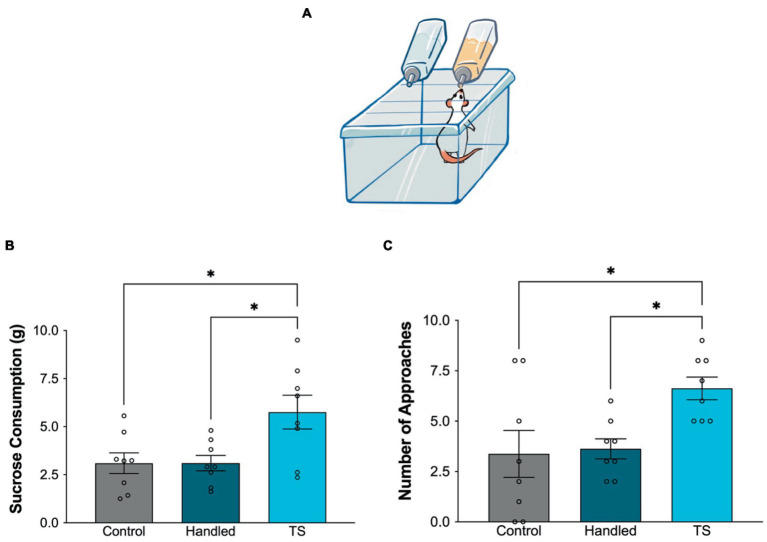
Behavioral evaluations in the sucrose consumption test in the control group, handled group and TS given WAG/Rij rats, **(A)** Simplified illustration of the sucrose consumption test. **(B)** Sucrose consumption in grams ^*^p = 0,0059 TS group compared to control, and handled WAG/Rij rats; **(C)** Frequency of approaching the bottle, ^*^p = 0,0238 TS group compared to control, and ^*^p = 0,0382 compared to handled WAG/Rij rats.

In the sucrose consumption test, the sucrose consumption of the control and handled group was statistically significantly lower than the TS group. Additionally, the frequency of approaching the bottle was found to be statistically lower in the control and handled group compared to TS group (Figure 3).

**Figure 4:**
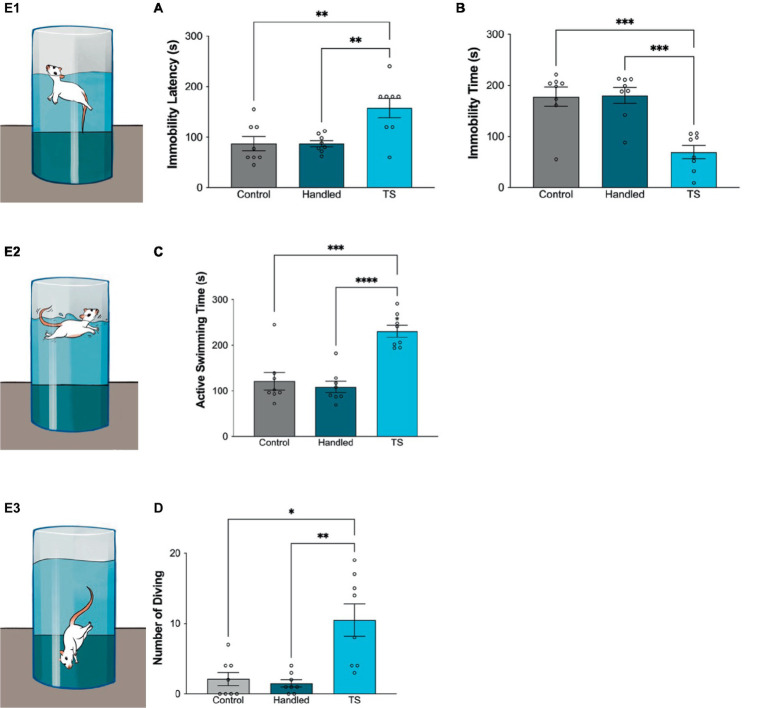
Behavioral evaluations in the forced swimming test in the control group, handled and WAG/Rij rats given TS, **(A)** Immobility latency, ^*^p = 0,0059 TS group compared to control, and ^*^p = 0,0059 compared to handled WAG/Rij rats; **(B)** Immobility time, ^*^p = 0,0125 TS group compared to control, and ^*^p = 0,0089 compared to handled WAG/Rij rats; **(C)** Active swimming time, ^*^p < 0.0001 TS group compared to control, and ^*^p = 0.0001 compared to handled WAG/Rij rats; **(D)** Number of diving, ^*^p = 0.0139 compared to control, and *p = 0.0074 compared to handled WAG/Rij rats. **E**_**1-3**_. Simplified illustrations of the forced swimming test.

Statistically significant differences were found in the forced swimming test amongst WAG/Rij rats given TS, control and handled group WAG/Rij rats. Immobility latency was longer in the group receiving TS, but immobility time was shorter. Additionally, active swimming time was found to be longer in WAG/Rij rats that received TS and the number of diving was statistically higher than in control and handled group rats (Figure 4).

### SWDs

**Figure 5:**
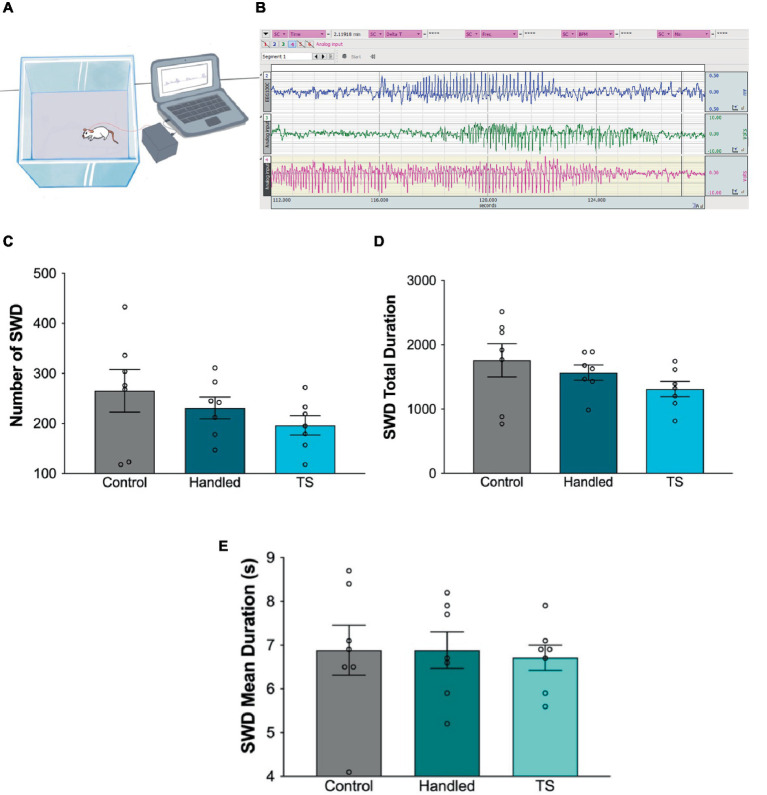
**(A)** Simplified illustration of the EEG recording. **(B)** Screenshot of the of EEG recordings. Total seizure duration **(C)**, number of SWDs **(D)** and mean duration **(E)** of the groups (*p* > 0.05).

Epileptic activity was evaluated by analyzing SWDs in EEG for 4 h in WAG/Rij rats given TS, handled and in the control groups. When the total duration, mean duration and number of SWDs were evaluated for 4 h, no statistically significant difference was found amongst the groups (*p* > 0.05). However, the total seizure duration and number of seizures in the tactile stimulation group in adulthood tend to decrease compared to the handled and control groups (Figure 5).

## Discussion

The interesting aspect of the results of this study was that tactile stimulation did not affect the development of absence epilepsy but reduced depression-like behaviors that are present in WAG/Rij rats.

The WAG/Rij rat absence epilepsy model is a suitable model for studying the development of epilepsy and comorbidities. WAG/Rij rats exhibit depression-like behavior compared to nonepileptic Wistar rats (Sarkisova et al., 2003; Sarkisova and van Luijtelaar, 2022; Sitnikova, 2024). Around 3 months, when SWDs become more evident, depression-like behavioral symptoms also emerge. As rats get older, absence epileptic seizures become more severe and their depression-like symptoms worsen (Sarkisova et al., 2014).

Depressive-like behavior is assessed using two distinct behavioral tests: the forced swimming test (FST) and the sucrose consumption test (SCT). Research has demonstrated that WAG/Rij rats exhibit longer periods of immobility in the FST and consume less sucrose compared to nonepileptic rats (Gruenbaum et al., 2021).

Our findings demonstrated that TS administration increased sucrose consumption and number of approaches to the bottle in the sucrose consumption test (SCT). Also, TS administration decreased immobility time and increased immobility latency, active swimming time and diving frequency in the FST.

The SCT has been developed taking into consideration the rats’ preference for sweets. Anhedonia, the main symptom of depression, can be assessed with the SCT (Kanner et al., 2012; Klein et al., 2015). Anhedonic behavior in rats can be assessed by providing them with simultaneous access to both tap water and sweetened solution. A healthy animal will have a preference for a sweet solution, but an anhedonic animal will drink a reduced amount of sweet solution compared to the control group. An important benefit of the SCT is that as the rats are held within cages similar to their home cages, the occurrence of stress or anxiety is minimal (Klein et al., 2015).

The FST is a commonly used experimental method for studying depression-like behavior. In this paradigm, a decrease in immobility duration and an increase in immobility latency, swimming time, and diving frequency are indicators of a positive effect of the intervention on depressive behaviors. This test evaluates the rats’ reaction to being forced to swim, which are: the duration of their swimming or struggle and duration of their immobility. The presence of increased immobility is considered as “hopelessness” or a “depressed mood,” (Crawley, 2007; Klein et al., 2015).

Studies have demonstrated that early drug treatments can reduce epilepsy and depression-like symptoms in WAG/Rij rats (Sarkisova et al., 2010; Russo et al., 2011; Sarkisova and van Luijtelaar, 2011; Russo and Citraro, 2021). In addition, in WAG/Rij rats, good maternal care given by nonepileptic Wistar mothers, has been shown to have disease modifying effect on epileptogenesis and comorbidogenesis (Sarkisova and Gabova, 2018).

Our previous study also showed that TS that carried out from postnatal days 3–21 affects the genetic absence epilepsy and comorbid depression-like behavior in the WAG/Rij rat model (Balikci et al., 2020). Many studies highlight a causal relationship between depression and absence seizures (Sarkisova et al., 2014; Sarkisova and van Luijtelaar, 2022), however, there is also contrary evidence. For example, Leo et al. (2019) reported that the suppression of absence epilepsy with drugs does not always counteract depressive-like behavior. Our study has shown that giving TS to young rats decreased only the depressive-like behaviors but did not affect the seizures.

Previous data and our results provide evidence that absence seizures and depressive-like behavior can originate from the same disease network, independently and separately (Mula and Sander, 2007; Leo et al., 2019).

Empirical research, both in clinical and experimental settings, has demonstrated that TS yields psychological benefits, including alleviation of depression and mitigation of mood disorders (Maruyama et al., 2012; Roversi et al., 2019; Costa et al., 2020).

TS’s effects on the brain and behavior are connected to many underlying mechanisms (Chu et al., 2021). Research indicates TS administration in adulthood has a positive impact on depression-like behaviors by influencing the hypothalamus-pituitary–adrenal (HPA) axis and regulating neurotrophic factors (Roversi et al., 2019; Costa et al., 2020). Depression has been associated with impaired functioning of the HPA axis (Plotsky et al., 1998; Barden, 2004; Pariante and Lightman, 2008; Guerry and Hastings, 2011). Glucocorticoids (GC) are produced and released by the adrenal glands when the HPA axis is active. The glucocorticoids are crucial in providing negative feedback to the HPA axis (Gjerstad et al., 2018). Changes in the GC receptors in cortico-limbic regions may contribute to depressive disorders by affecting the release of glucocorticoids. Furthermore, heightened secretion of GCs may have a detrimental effect on neurotrophic factors like BDNF (Numakawa et al., 2017). This can result in a decline in brain synaptic plasticity, which is one of the potential ways that GCs contribute to depression, as shown by Roversi et al. (2019).

Costa et al. (2020) showed that TS effectively decreased the release of corticosterone and epinephrine in response to stress and lowered norepinephrine levels. Then, they proposed that TS to adult animals might regulate the HPA axis and the sympathetic nervous system, resulting in decreased adrenal responses and reduced basal activity of sympathetic nerves.

Additionally, another study demonstrated that TS improves symptoms of depression by positively influencing the signaling of the HPA axis (Roversi et al., 2019). This is evident in the changes observed in adrenal weight, release of corticosterone, and alterations in the expression of glucocorticoid receptors. Furthermore, TS enhances the levels of neurotrophic factors in the cortex, as reported by Roversi et al. (2019).

Taken together, it can be suggested that TS ameliorated depression-like behaviors in adult WAG/Rij rats, through its influence on HPA axis signaling which may lead to modulation of neurotropic factors, enhancing the neural plasticity for long lasting effects on depression.

Furthermore, various studies have indicated that the TS’s effects are linked to an increase in the release of dopamine (DA) and/or serotonin in the brain. Clinical studies have demonstrated an increase in the concentration of DA and serotonin in urine following massage therapy (Field et al., 2005; Mueller and Grunwald, 2021). A previous study demonstrated that a 5-min application of tactile stimulation to the back resulted in a considerable increase in DA release in the nucleus accumbens (NAc) of both conscious and anesthetized rats (Maruyama et al., 2012).

WAG/Rij rats with depression-like behavior exhibit impaired functioning in the brain’s dopamine system, as evidenced by behavioral, electrophysiological, and pharmacological studies (Birioukova et al., 2005; Sarkisova et al., 2008; Fedosova et al., 2023). It has been shown that a decrease in the activity of the dopamine system in the mesolimbic region of the brain is linked to both absence seizures and depression-like symptoms in WAG/Rij rats (Birioukova et al., 2005; Sarkisova et al., 2022; Fedosova et al., 2023). Administering mixed dopamine D1/D2 receptor agonists systemically led to a decrease in SWDs and comorbidity with depression-like symptoms in WAG/Rij rats (Midzianovskaia et al., 2001; Sarkisova et al., 2023). Conversely, dopamine antagonists enhanced the occurrence of SWDs and depression-like behavioral symptoms in these rats (Sarkisova et al., 2008, 2023). The basal ganglia regulate absence seizures. The transmission of DA neurotransmitters in both the dorsal and ventral striatum has an important role (Midzianovskaia et al., 2001; Sarkisova et al., 2023).

The ventral striatum, particularly the NAc, plays a crucial role in regulating both absence seizures and depression-like behavioral symptoms in WAG/Rij rats (Sarkisova et al., 2003; van Luijtelaar and Zobeiri, 2014; Sarkisova et al., 2023; Tsyba et al., 2023a,b). Decreased dopaminergic activity in the striatum is thought to cause predisposition to cortical hyperexcitability and epilepsy (Sarkisova et al., 2003).

Recent biochemical investigations have demonstrated a decrease in DA levels in the NAc and striatum of WAG/Rij rats (Kiu et al., 2013; Birioukova et al., 2016; Fedosova et al., 2023; Sarkisova et al., 2023; Tsyba et al., 2023a,b).

It is also possible that the beneficial influence on depression-like behaviors of TS can be related to an increase in DA release in the brain during development and DA-dependent neural plasticity (Field et al., 2005; Mueller and Grunwald, 2021; Rossato et al., 2022).

On the other hand, depression-like behaviors decreased in WAG/Rij rats treated with TS, we did not find any change in seizure activity.

There are several studies showing that interventions very early in life (P3-P21) can alter the development of absence seizures later in life (Kovács et al., 2012; Balikci et al., 2020). However, studies demonstrate that the effects of interventions starting between P21 and P45 on seizures are generally temporary (Russo and Citraro, 2018). It can be argued that the onset time/age of the intervention is important for disease-modifying effects. Moreover, it is suggested that silent period (before the age of P50- P60) offers a range of opportunity in which an appropriate treatment could prevent or modify epileptogenesis (Leo et al., 2019). Our data indicate that TS treatment starting during late adolescence was not able to exert long-lasting disease-modifying effects at WAG/Rij rats. Based on this background, it could be considered that TS starting after the silent period may not have a modifying effect on epileptogenesis in WAG/Rij rats.

In the future studies, it would be interesting to investigate the effects of TS in epilepsies and comorbidities of adult WAG/Rij rats, and gender differeneces.

## Conclusion

Here we demonstrate that TS induces a comorbidity-modifying effect in the WAG/Rij rat model of generalized absence epilepsy. TS at late adolescence and early adulthood mitigates depression-like behavioral phenotype in WAG/Rij rats. The present data support the notion that environmental manipulations may affect epileptogenesis and comorbidogenesis. The administration of TS during adolescence, a crucial stage for the development of psychiatric disorders, may provide long-lasting protection against depression.

## Data availability statement

The original contributions presented in the study are included in the article/supplementary material, further inquiries can be directed to the corresponding author.

## Ethics statement

The animal study was approved by University of Kocaeli Animal Ethics Committee. The study was conducted in accordance with the local legislation and institutional requirements.

## Author contributions

AB: Methodology, Writing – original draft, Writing – review & editing. UE: Data curation, Methodology, Writing – original draft. VG: Data curation, Software, Writing – original draft. GI: Conceptualization, Formal analysis, Methodology, Writing – original draft, Writing – review & editing.
